# Parent-Reported Rate of the Use of Antibiotics in Children: A Cross-Sectional Study

**DOI:** 10.7759/cureus.32720

**Published:** 2022-12-20

**Authors:** Hamdan N Alajami, Abdullah M Saeed, Abdullah N Aldosari, Azzaz M Alkharan, Fatima A Lubbad, Hind M Almutairi, Nuha N Mazeed, Samah S Alwallan, Jasser A Alzhrani

**Affiliations:** 1 Pharmaceutical Care Services, King Saud Medical City, Riyadh, SAU; 2 Pharmaceutical Care, East Jeddah General Hospital, Jeddah, SAU

**Keywords:** antibiotic therapy, parental, parent survey, irrational antibiotic use, antibiotic use

## Abstract

Background

Antibiotic resistance is growing worldwide due to the magnitude of the rational and irrational use of antibiotics, particularly in children. Evidence regarding the use of antibiotics without a prescription in Saudi children is limited, and the factors that affect frequent antibiotic use in children are poorly understood. Therefore, we investigated the rate of the use of antibiotics in Saudi children reported by their parents and the factors associated with parents that affect the use of antibiotics in children.

Methods

A cross-sectional survey using a 27-item self-administered questionnaire was conducted among parents living in Saudi Arabia. Parents with at least one child aged 16 years or less were eligible to participate in the study. The results were analyzed via descriptive and inferential statistics.

Results

A total of 284 parents participated in the study. Of the participants, 81% (n = 230) had given their children at least one course of antibiotics, and 57% of their children were male (n = 164). Many parents did not have a regular general practitioner (GP) for providing care to their children (n = 201, 70%). Further, 164 (n = 71%) parents administered antibiotics without consulting a general practitioner. Neither the parent and child demographics nor the parent knowledge and behavioral variables were significantly associated with the parent’s variable of interest in the administration of antibiotics.

Conclusions

Generally, parents reported alarmingly high rates of antibiotic use among their children. Reducing the unnecessary use of antibiotics in children is crucial for preventing antimicrobial resistance. No apparent statistically significant factor was identified as being associated with antibiotic use. The need for additional measures to limit antibiotic use in children may be warranted. Initiatives to educate parents for consulting a regular general practitioner for their children before administering antibiotics may improve the health outcomes of children.

## Introduction

Globally, antibiotic resistance is increasing predominantly and a public health threat due to the magnitude of rational and irrational antibiotic use, particularly in children [1-4]. Previous studies reported that irrationality or the overuse of antibiotics to treat viral respiratory tract infections is one of the key reasons for antibiotic resistance [5-8]. In 2015, more than two-thirds (44%) of the Australian people, including half of the children aged 14 years and less, were prescribed at least one systemic antibiotic [2]. Evidence indicates that irrational antibiotic use is also associated with cultural factors, behavioral aspects (e.g., self-medication and socioeconomic status), and level of education [9]. Moreover, lack of education regarding health is one of the major factors that contribute to irrational antibiotic use [10].

Globally, two-thirds of all oral antibiotics are consumed without a prescription and are inappropriately used particularly for mild childhood infections, malaria, tuberculosis, and pneumonia. Similarly, this practice is also comparable in Saudi Arabia [11,12]. The overall impact of irrational antibiotic use not only affects the patients but also the community because of increasing antibiotic resistance, which is an alarming crisis in Saudi Arabia [13]. The population aged 14 years and less was about 26% in Saudi Arabia [14], and therefore, they likely need care from their parents during their diseases.

Evidence regarding the use of antibiotics without a prescription in Saudi children is limited, and the factors that affect frequent antibiotic use in children are poorly understood. Previous studies primarily focused on the awareness and attitude using a different tool that lacked specific questions on the frequency of the use of antibiotics and factors associated with parents that may affect regular antibiotic use in Saudi children [15,16]. Therefore, we aimed to investigate the parent-reported rate of the use of antibiotics in Saudi children and the factors (e.g., behaviors, knowledge, and attitudes) associated with parents that affect the use of antibiotics in children. This evidence is crucial for policymakers to develop strategies to reduce the impact of irrational antibiotic use in Saudi Arabia.

## Materials and methods

Study design and setting

We conducted an online cross-sectional survey in October 2022 among parents in Saudi Arabia.

Inclusion and exclusion criteria

We only recruited those Saudi parents with at least one child aged 16 years or less and excluded parents without children. Only one parent per household was allowed to complete the questionnaire.

Data collection

The study researchers collected anonymous data from the parents using an online questionnaire developed through Google Forms. A 27-item self-administered questionnaire was distributed among parents living in Saudi Arabia (Appendices). All questions for the study tool were adapted from a previous study [17]. The face validity of the questionnaire was determined via a pilot sample of 10 parents. For content validity, the study tool was also reviewed based on the piloting feedback to ensure that it is easy to comprehend and complete. The study tool comprised questions regarding sociodemographic information (e.g., age, gender, level of education, employment status, and socioeconomic status), consumption of antibiotics in children, and parental factors including behaviors, knowledge, and attitudes that affect the use of antibiotics in children. For the recruitment of participants, we applied multiple recruitment strategies such as social media (e.g., Twitter and Facebook) and direct contact with the parents who visited shopping malls and healthcare settings.

Ethical considerations

Two study researchers briefed the parents about the purpose of the study and provided a study information sheet. The ethics committee granted a waiver for the written informed consent before the initiation of the study. The study was initiated after obtaining ethical permission from the Institutional Review Board of King Saud Medical City (approval number: H1RI-30-22-04) and conducted according to the principles of the Declaration of Helsinki. The Strengthening the Reporting of Observational Studies in Epidemiology statement was followed for reporting this study [18].

Sample size and statistical analysis

Based on literature evidence, 52% of parents administer antibiotics to at least one of their children [17]. Given this proportion, a sample size of 194 was estimated using an online calculator [19]. We compared the demographics (e.g., parent and child) and knowledge and behavior of parents related to antibiotic use between parents who had already given antibiotics to their children during the last 12 months and those who had not. We followed the recommendations of the US Centers for Disease Control and Prevention for the correct responses about the indication for antibiotics. For analyzing the data, the option “uncertain” was considered equivalent to “disagree.” We used binary logistic regression to examine associations between variables related to parents with the administration of antibiotics to at least one child. A binary logistic model was utilized for the outcome of the provision of antibiotics. Further, a Poisson model for count data was assumed for the number of antibiotic courses that were given to children. We also calculated the effect estimates and associated 95% confidence intervals (CIs). All analyses were declared significant at p < 0.05. All statistical analyses were performed using the Statistical Package for the Social Sciences (SPSS) software version 25.0 (IBM SPSS Statistics, Armonk, NY, USA).

## Results

Table 1 shows the characteristics of the respondents. There were primarily females (58%) and young adults between the ages of 31 and 40 years (42%) in the sample. Over half (57%) of them were bachelor’s degree holders. A little less than two-thirds (63%) of the participants were employed full time. Only roughly a third of them (36%) had household incomes above 15,000 Saudi Riyal (SR) monthly. Of the participants, 71% did not have a dedicated primary care practitioner. Further, 81% of the participants had given their children at least one course of antibiotics, and 57% of their children were male. Many parents did not have a regular general practitioner (GP) for providing care to their children (n = 201, 70%). Further, 164 (n = 71%) parents administered antibiotics without consulting a general practitioner.

**Table 1:** Characteristics of the parents SR: Saudi Riyal

Characteristics	All parents (number)	Parents involved in providing antibiotics (number (%))
Total	284	230 (81%)
Gender		
Male	127	97 (42%)
Female	155	133 (58%)
Age		
18-30	57	44 (19%)
31-40	121	95 (41%)
41-50	74	65 (28%)
51-60	10	9 (4%)
61+	2	2 (1%)
Highest education level		
Middle school	9	8 (3%)
High school	51	42 (18%)
Bachelor’s degree	161	130 (57%)
Master’s degree	32	28 (12%)
PhD degree	3	2 (1%)
Other	15	11 (5%)
Employment status		
Working full time	184	144 (63%)
Working part time	15	14 (6%)
Unemployed, not currently seeking work	12	12 (5%)
Unemployed, currently seeking work	21	15 (7%)
Home duties	41	37 (16%)
Retired	8	7 (3%)
Permanently unable to work	1	1 (0%)
Approximate household income		
Less than 10,000 SR	77	63 (27%)
From 10,000 to 14,999 SR	93	79 (34%)
From 15,000 to 19,000 SR	64	51 (22%)
More than 20,000 SR	41	33 (14%)
Do you have a regular general practitioner for your child?		
Yes	80	65 (28%)
No	201	164 (71%)
Gender of child		
Male	164	131 (57%)
Female	114	98 (43%)

Potential predictors of antibiotic use in the previous 12 months were given in Table 2. Neither the parent and child demographics nor the parent knowledge and behavioral variables were significantly associated with the parent’s variable of interest in the administration of antibiotics.

**Table 2:** Predictors of antibiotic use in the preceding 12 months OR: odds ratio, CI: confidence interval, GP: general practitioner

Variable	OR (95% CI)
Demographics (parents) (n = 284)	
Older age, years	1.917 (0.838-4.386)
Job status	0.534 (0.229-1.245)
Income	1.284 (0.497-3.317)
Have a regular GP for child healthcare	
Male	0.861 (0.422-1.761)
Older child age, years	1.020 (0.204-5.105)
Parent knowledge and behavior (antibiotics indicated, yes)	
Bacterial infections	0.912 (0.711-1.169)
Runny nose	1.410 (0.478-4.160)
Green nasal discharge	1.020 (0.636-1.636)
Sore throat	1.458 (0.831-2.558)
Ear infection	1.340 (0.973-1.845)
Viral infections	1.225 (0.756-1.987)
Cold	0.649 (0.391-1.077)
Influenza	0.848 (0.538-1.337)
Diarrhea	0.706 (0.387-1.289)
Urinary tract infections	0.810 (0.605-1.085)
Indication questions	
Heard of antibiotic resistance	1.228 (0.346-4.357)
Believe that antibiotic resistance is a worldwide problem	0.634 (0.335-1.199)
Believe that antibiotic resistance would have an impact on you or your family	1.470 (0.824-2.624)
Would take my child to another doctor	0.892 (0.669-1.188)

## Discussion

This study aimed to assess the rate of antibiotic use among Saudi children as reported by their parents and identify the parental characteristics that influence antibiotic use among their children. Four in five parents had administered at least one course of antibiotics to their children. Parental and child demographics, as well as parental knowledge and behavior, were not statistically linked with antibiotic use.

Although we did not uncover predictors of antibiotic usage in our study, presumably due to the relatively small sample size, there is much evidence in the literature reporting certain factors influencing the rate of antibiotic use. For instance, a previous study that used a relatively larger sample size (i.e., 2,157) identified poor parental knowledge of antibiotics as a predictor of antibiotic use, whereas our sample is smaller, which precludes the observance of statistically significant factors affecting antibiotic use [20]. Irrational antibiotic use may also be influenced by parents’ poor socioeconomic status, low education, and certain behavioral factors, such as self-medication use practice [9,10,17,20].

Antibiotic resistance is a major problem in Saudi Arabia, affecting not only patients but the general public [13]. Increased antibiotic use increases the risk of misuse and antibiotic resistance [17]. In addition, antibiotic resistance is a serious and growing problem, with widespread reports of its occurrence worldwide [21,22]. An overreliance on antibiotics is one of the leading causes of bacteria that are resistant to these drugs. The global approach to combat antimicrobial resistance may require a decrease in the inappropriate use of antibiotics among children.

Educational interventions aimed at parents and physicians have been shown to improve parents’ knowledge and understanding of antibiotic indications and antibiotic resistance [23]. The distribution of educational online tools about when and how to administer antibiotics is an effective strategy [24]. Antibiotic resistance is typically the result of improper or excessive antibiotic usage, which can be prevented with an effective and comprehensive education campaign. Publicly visible posters urging prudent antibiotic use may help curb the misuse of antibiotic use. Antibiotic stewardship programs have also emerged as a valuable tool for controlling the spread of antimicrobial resistance and should be suggested and implemented in all areas where antibiotics are routinely used. A further crucial step in preventing antibiotics misuse is to focus on the currently available antibiotics with updated, targeted guidelines.

This study has some limitations. Current parents’ behaviors, knowledge, and attitudes are reflected in our research. Although it would be useful to understand how parents’ behaviors, knowledge, and attitudes evolve over time, which was not covered in our study, this is a potentially lucrative field for future research if longitudinal approaches are employed. Knowing what was meant by terms such as “viral” and “bacterial” was crucial for measuring familiarity with antibiotics in our study, although several of the terms given in the questionnaire were not described in greater detail. Since we did not record the antibiotic prescribed, the underlying condition, or the length of treatment, we were unable to determine whether or not antibiotic use was appropriate, another potential future research area.

## Conclusions

Generally, parents reported alarmingly high rates of antibiotic use among their children. Reducing the unnecessary use of antibiotics in children is crucial for preventing antimicrobial resistance. No apparent statistically significant factor was identified as being associated with antibiotic use. The need for additional measures to limit antibiotic use in children may be warranted. Initiatives to educate parents for consulting a regular general practitioner before administering antibiotics may improve the health outcomes of children.

## Appendices

The 27-item self-administered questionnaire used in the present study is shown in Table 3.

**Table 3:** Study questionnaire SR: Saudi Riyal, GP: general practitioner

Questionnaire	
What is your age (in years)?	18-30
	31-40
	41-50
	51-60
	61 and above
What is your gender?	Female
	Male
Highest education level	Middle school
	High school
	Bachelor’s degree
	Master’s degree
	PhD degree
	Other: ___________________
Employment status	Working full time
	Working part time
	Unemployed, not currently seeking work
	Unemployed, currently seeking work
	Home duties
	Retired
	Permanently unable to work
Approximate household income	Less than 10,000 SR
	From 10,000 to 14,999 SR
	From 15,000 to 19,000 SR
	More than 20,000 SR
Do you have a regular GP who provides care to your child?	Yes
	No
What is the age of your child?	
What is the gender of your child?	Female
	Male
In the past 12 months, how many times has your child taken a course of antibiotics?	One time
	Two times
	Three times
	Four times
	>4 times
	None
If your child took oral antibiotic courses in the last 12 months, did you give to your child before or without seeing a doctor?	Yes
	No
	Not applicable (the child did not take an oral antibiotic course in the last 12 month)
Where did you get the oral antibiotics that you started giving to your child before or without seeing a doctor? Please select all that apply.	The antibiotics were left over from a previous prescription
	The antibiotics were given to me by a family member or friend
	The antibiotics were purchased online without a prescription
	The antibiotics were purchased from a pharmacy without a prescription
	Other: ___________________
Did you stop before the full antibiotic course was completed?	Yes
	No
	Not applicable (an oral antibiotic course was not given to my child in the past 12 months)
For what reasons did you stop giving the antibiotics? Please select all that apply.	My child was having side effects from the antibiotics
	My child’s symptoms had gone away
	The antibiotics did not seem to be working
	My child refused to take the antibiotics
	Other: __________________
Antibiotics are needed to treat viral infection	Definitely no
	No
	Uncertain
	Yes
Antibiotics are needed to treat bacterial infection	Definitely no
	No
	Uncertain
	Yes
Antibiotics are needed to treat runny nose	Definitely no
	No
	Uncertain
	Yes
Antibiotics are needed to treat green nasal discharge	Definitely no
	No
	Uncertain
	Yes
Antibiotics are needed to treat sore throat	Definitely no
	No
	Uncertain
	Yes
Antibiotics are needed to treat ear infection	Definitely no
	No
	Uncertain
	Yes
Antibiotics are needed to treat colds	Definitely no
	No
	Uncertain
	Yes
Antibiotics are needed to treat influenza or flu	Definitely no
	No
	Uncertain
	Yes
Antibiotics are needed to treat diarrhea	Definitely no
	No
	Uncertain
	Yes
Antibiotics are needed to treat urinary tract infection	Definitely no
	No
	Uncertain
	Yes
If I believed my child needed an antibiotic but my doctor did not prescribe one, I would take my child to another doctor	Strongly agree
	Agree
	Neither agree nor disagree
	Disagree
	Strongly disagree
Have you heard of the term “antibiotic resistance”? Antibiotic resistance is the ability of bacteria to resist the effects of antibiotics, that is, the germs are not killed, and their growth is not stopped. Infections with antibiotic-resistant bacteria are difficult to treat.	Yes
	No
	Not sure
Antibiotic resistance is a big problem worldwide	Strongly agree
	Agree
	Neither agree nor disagree
	Disagree
	Strongly disagree
I am worried about the impact that antibiotic resistance will have on my health and that of my family	Strongly agree
	Agree
	Neither agree nor disagree
	Disagree
	Strongly disagree
